# Perspective on oral medication adherence among patients with acute graft-versus-host disease: a qualitative descriptive study

**DOI:** 10.1007/s00520-024-08825-4

**Published:** 2024-09-04

**Authors:** Chiara Visintini, Chiara Lucchetta, Margherita Venturini, Irene Mansutti, Stefania Chiappinotto, Francesca Patriarca, Alvisa Palese

**Affiliations:** 1 Department of Biomedicine and Prevention, University of Rome Tor Vergata, Montpellier’s Street, 1, 00133 Rome, Italy; 2 Haematology and Stem Cell Transplantation Unit, Udine University Hospital, Azienda Sanitaria Universitaria Friuli Centrale, Udine, Italy; 3 Oncology Unit, Udine University Hospital, Azienda Sanitaria Universitaria Friuli Centrale, Udine, Italy; 4 Department of Medicine, University of Udine, Udine, Italy

**Keywords:** Graft-versus-host disease, Haematopoietic stem cell transplant, Immunosuppressants, Medication adherence, Patient perspective

## Abstract

**Purpose:**

Despite the importance of adherence to immunosuppressants (IMMs) after an allogeneic haematopoietic stem cell transplant (HSCT) for the treatment of acute graft-versus-host disease (aGvHD), no studies to date have reported the experiences of such patients concerning medication adherence (MA). Therefore, the aim of the study was to explore the perspective on MA to immunosuppressive oral therapy among allogeneic HSCT patients with aGvHD.

**Methods:**

A qualitative descriptive study following a reflexive thematic analysis methodological approach was performed involving a purposive sample of 16 patients with aGvHD who were being cared for in the outpatient setting of a bone marrow transplant centre and were willing to participate. Semi-structured audio-recorded interviews were conducted, transcribed verbatim and thematically analysed; member checking was performed. COnsolidated criteria for REporting Qualitative research (COREQ) and the ESPACOMP Medication Adherence Reporting Guideline were followed.

**Results:**

Participants aged 25–74 years and mostly males (62.5%) were recruited for this study; 56.2% developed grade I, 37.5% grade II and 6.3% grade III aGvHD; 56.2% were receiving treatment with both cyclosporine and prednisone. Patients' perspectives have been summarised into four themes, named: “Transiting from an external obligation to a habit”; “Being in the middle between the negative and positive effects of the IMMs”; “Failure to systematically respect the rules”; and “Adopting personal strategies to become adherent”. After difficulties with the perception of feeling obliged, patients became used to adhering to IMMs. Although there were failures in systematically taking the medication correctly and there were episodes of non-adherence, the adoption of personal strategies helped patients to become adherent to their medication schedules.

**Conclusions:**

MA in patients with aGvHD is a complex behaviour and is often a challenge. These results can help healthcare professionals and centres to understand how best to design tailored strategies and behavioural interventions to maximise patients’ MA to IMMs.

**Supplementary Information:**

The online version contains supplementary material available at 10.1007/s00520-024-08825-4.

## Introduction

A total of 46,143 haematopoietic stem cell transplants (HSCTs) were reported by 689 European centres in 2022, 41.2% of which were allogeneic [1]. For at least the first six months after HSCT, patients need to adhere to their treatment with immunosuppressants (IMMs) to prevent and treat graft-versus-host disease (GvHD) [2], which is one of the most frequent allogeneic HSCT-related complications, next to disease relapse (up to 40% for some diseases) [3] and bacterial (20–50%), viral (15–30%) and fungal (6–8%) infections [4].

GvHD is an immune-mediated reaction based on a physiopathological mechanism triggered when the immune T cells in the donated tissue (the graft) recognise the recipient (the host) as unfamiliar; the new immune response activates donor T cells by promoting cytolytic activity that attacks the recipient’s tissues to eliminate foreign antigens [5]. Acute GvHD (aGvHD) usually occurs in the first period after allogeneic HSCT, during hospitalisation or after discharge; it has an incidence of 30–50% [6, 7] and leads to reduced overall survival [8] with mortality rates close to 10.7% [9]. The grading system of aGvHD considers the involvement of the skin (maculopapular rash and up to generalised erythroderma), the gastrointestinal tract (anorexia, nausea, vomiting, diarrhoea and abdominal pain), and the liver (hyperbilirubinemia), resulting in four possible degrees of aGvHD (I–IV) [10]. On the other hand, chronic GvHD (cGvHD), with an incidence of 20–30% [7, 9], involves more anatomical structures, especially after hospital discharge: the skin, mouth, eyes, lungs, gastrointestinal tract, liver, genitalia and joints can be involved in cGvHD with a severity score ranging from mild to severe [11]. Steroids and cyclosporine A are the cornerstones of first-line aGvHD therapy [2, 12]; however, negative effects consequent to this therapy are reported, such as tremor, skin rash, head and stomach ache, weight gain, change of taste or neurological pain [13, 14]. Although IMMs are required to manage aGvHD and to decrease its risk in evolution to the chronic form, hospital readmissions and mortality [15–18], it is not always possible to prevent the onset of aGvHD. Moreover, patients with a molecular relapse might need to stop or taper IMMs earlier, and physicians accept the risk of GvHD rather than disease mortality [2].

Both forms of GvHD affect patients’ quality of life (QoL) [19]. However, despite the relevance, few studies on patients’ experiences have been reported to date; those published have mostly focused on cGvHD, reporting visible transformations and side effects of long-term corticosteroid treatments that trigger a sense of embarrassment and distress, due partly to their intermittent nature [20, 21]. Moreover, to our best knowledge, no qualitative data have been published on the experiences of patients with aGvHD with respect to medication adherence (MA), except for a pilot trial [22] documenting the patient-reported outcome (PRO) measures regarding QoL. Fatigue, decreased appetite, problems tasting, loose stools, pain, itching and depression were the most prevalent symptoms among patients with grade II–IV aGvHD, as measured with the PRO Common Terminology Criteria for Adverse Events (CTCAE) [22].

MA is a complex behaviour defined as “the process by which patients take their medications as prescribed”, composed of three steps: *initiation*, *implementation* and *persistence* [23]. *Initiation* is the beginning of the prescribed medication intake; *implementation* is the correspondence between the patient’s actual dosing and the medical prescription, from initiation until the last dose is taken. Finally, *persistence* is the time between the first and the last intake of the prescribed medication [23]. Non-adherence to medications occurs when there is late or non-initiation of the prescribed treatment, sub-optimal implementation of the dosing regimen, or early discontinuation of the treatment [23].

MA to immunosuppressive therapy has been documented as poor among HSCT patients (around 61.5% [17]). Few studies have addressed the relationship between MA to immunosuppressive oral therapy and GvHD. A Spanish retrospective study [16] evaluating MA in 46 patients concluded that 84.8% of the patients were adherent and the incidence of aGvHD in the non-adherent was 55.6%, compared to 45.9% in adherent patients (odds ratio [OR] 0.68; 95% confidence interval [CI] 0.157–2.943; *p*-value = 0.718). In a second French study [24], among 33 patients, 54.6% were poorly adherent, of whom 38.9% developed aGvHD. Among adherent patients, only 26.7% developed a form of aGvHD, although in this study, too, the results were not statistically significant (*p* = 0.71). Regarding the chronic form of GvHD, medication non-adherence (MNA) has been documented as higher among patients with mild cGvHD than among patients without cGvHD (OR 2.63; 95% CI 1.04–6.66; *p* = 0.042), with a trend towards significance also seen in moderate cGvHD (OR 2.58; 95% CI 0.91–7.34; *p* = 0.076) [25].

Among the recipients of HSCT, factors that could contribute to reduce MA are younger age, high levels of distress and psychosocial risk (e.g. psychiatric symptoms, substance use, transplant knowledge, social supports and lifestyle factors), lack of support from caregivers [17], and psychological issues (e.g. beliefs about medicines and health locus of control) [26]. Even an increase in the number of daily doses of IMMs or a decrease in the number of concomitant medications are factors increasing MNA [17]. From the solid organ transplant literature, emesis or nausea, wrong information, lack of routine, longer time since transplantation, forgetfulness and not having medicines when away from home are all barriers to MA [27–29]. The question of how to promote MA in the context of allogeneic HSCT [30, 31] has been investigated in four qualitative studies, including 44 patients in total [32]. Findings showed that when patients implemented their medication management at home, they recognised the importance of tracking time every day, because every few hours there was the need to take medications. Beyond MA obstacles—both internal (e.g. lack of knowledge, emotional issues) and external to patients (e.g. medication effects)—strategies promoting MA are self-based or rely on other resources such as healthcare professionals (HCPs), caregivers, and digital technologies, as has been reported recently [33].

However, evidence specifically regarding patients with aGvHD is not available. Therefore, with the aim of expanding the available knowledge in this field and planning new strategies to support MA to IMMs, the aim of this study was to explore the perspective on MA to immunosuppressive oral therapy among allogeneic HSCT patients with aGvHD.

## Methods

### Study design

A descriptive qualitative study was performed following a reflexive thematic analysis methodological approach, an independent qualitative descriptive approach as “a method for identifying, analysing and reporting patterns (themes) within data” [34]. The study is reported in accordance with the COnsolidated criteria for REporting Qualitative research checklist [35] (Supplementary Table 1) and the ESPACOMP Medication Adherence Reporting Guideline (EMERGE) [36] adapted to the study design.

### Participants and setting

Participants were purposively selected [37] among those attending the outpatient setting of an academic bone marrow transplant centre in the north-east of Italy, where on average 70 allogeneic HSCTs take place per year and the management of around 30–40 aGvHD cases per year is documented. Eligible patients were as follows: (a) aged ≥ 18 years; (b) diagnosed with aGvHD after allogeneic HSCT; (c) requiring oral IMMs (at least one) during the *implementation* phase [23]; and (d) willing to participate in the study. We excluded patients who were as follows: (a) experiencing chronic GvHD; (b) not able to understand and speak the Italian language; or (c) in poor clinical condition, to prevent any additional pressure.

Patients were asked to participate in this study during their medical follow-up between September 2023 and February 2024, after being informed of the study’s aims and procedures by the researcher affiliated with the transplant centre (C.V.). No patients declined the invitation to participate, and all gave informed consent. The patients’ involvement ended when data saturation had been achieved [38], as judged independently by three researchers (C.V., C.L. and I.M.).

### Study context

The first-line treatment for aGvHD is administered for ≥ grade II [2, 12], with a starting dose of 2 mg/kg/day 6-methylprednisolone intravenously (in hospitalised patients) and 2.0–2.5 mg/kg/day prednisone (after discharge) for at least seven days. The tapering of steroids depends on the aGvHD response: in cases of complete response, the steroid dose is gradually reduced over a period of one month. In cases of steroid-refractory aGvHD, we start a second-line therapy with 20 mg/day ruxolitinib orally or extracorporeal photopheresis (ECP) in patients with active infections or severe cytopenia. We use topical steroids (hydrocortisone butyrate 0.1% or clobetasol butyrate 0.05%) for aGvHD grade I stage 1–2 skin. Non-absorbable oral steroids, such as budesonide (9 mg/day) are given in addition to systemic corticosteroids for gastrointestinal aGvHD. Cyclosporine concentration is carefully monitored twice a week to avoid toxicity. The values of therapeutic drug monitoring (TDM) from blood samples are used to adapt the cyclosporine dosage; we consider the range 100–250 ng/mL as a target until the third month after HSCT.

Cyclosporine A is also used for the prevention of aGvHD in our centre, in addition to post-transplant cyclophosphamide and oral mycophenolate mofetil or rabbit anti-thymocyte globulins and methotrexate, depending on the type of allogeneic HSCT performed. The duration of cyclosporine prophylaxis is six months; however, it will be adjusted due to the risk of disease relapse, chimerism and presence of aGvHD. If no aGvHD is reported, it is tapered from four months after HSCT until it stops. In the case of an early molecular relapse, physicians stop or taper IMMs in advance, taking the risk of aGvHD, benefiting of the related effect of the graft-versus-tumour.

Any systematic adherence supporting intervention is currently performed in our medication management before hospital discharge. Usually, oral cyclosporine A and prednisone are administered by the nurses in the last days of hospitalisation (*initiation* [23]), where the nurses observe the intake very carefully. Meanwhile, the patients are trained to take cyclosporine within a maximum of 30 min before or after the correct time. Moreover, patients are educated by nurses as to what GvHD is and to avoid the intake of grapefruit and its derivatives, *Hypericum perforatum* and some side effects (e.g. tremor, weight gain, steroid-induced diabetes). However, this is not a structured educational intervention, and every nurse could provide it differently. Finally, at discharge, the physician shares with the patient the discharge letter containing the list of medications to be taken at home, including IMMs, with dosage and time.

Criteria for defining a patient as non-adherent to IMMs in our centre during the *implementation* [23] are episodes of non-taking (missing at least one dose), mistakes (unintentionally taking one medication instead of another), or delays (time deviation of more than 30 min after or before the right time). Patients are asked during follow-up visits if they have faced these described episodes and their responses being empirically used to measure adherence to cyclosporine A. Thus, no tools were used to measure MA during the conduct of the study.

### Data collection

Data were collected through face-to-face, semi-structured interviews. The topics of the interview guide were developed by the research team after consulting the literature [30–33, 39] and according to their professional haematological backgrounds (C.V., M.V.) to combine deductive evidence-based and inductive approaches. The topics were: participants’ perceptions and knowledge regarding immunosuppressive oral therapy; thoughts while taking IMMs and at treatment initiation; implications of medications for daily life (e.g. side effects); reminders of an episode of MNA; and strategies used to ensure MA. Participants who developed aGvHD after discharge were asked three open-ended questions, whereas participants who developed aGvHD during hospitalisation were asked a single question (Supplementary Table 2). Demographic and clinical data were collected (e.g. age, sex, date of onset and stage [10] of aGvHD, response to IMMs at the time of interview, IMM medications) by accessing the electronic medical records. All questions were tested in pilot interviews for clarity and feasibility with two participants identified using the same inclusion/exclusion criteria: no changes were found to be necessary.

A relationship was established with participants prior to study commencement and the involved researchers explained their role and the study’s aims and procedures to participants. Researchers shared their pre-conceptions regarding the phenomenon under study in a meeting [40] before starting the interviews to make these visible and to reduce their influence during the study process.

After collecting written consent from participants, the interviews were performed and audio recorded. The first five interviews were conducted alternately by two female team members (C.V., M.V.) with haematological backgrounds and prior experience of conducting qualitative interviews, with a student in training in Nursing Studies (C.L.) present. From the sixth interview onwards, the third researcher (C.L.) conducted the interviews under the supervision of C.V. and M.V. Field notes were collected during all interviews, which were performed in a private room at the outpatient haematological centre, allowing participants privacy and comfort. As one participant wished, one interview was conducted with his caregiver. Interviews lasted approximately 13 to 48 min.

### Data analysis

Audio recordings were transcribed verbatim by one researcher (C.V.) within 48 h and each participant being assigned a code (e.g. P1, P2) to protect their identity. A thematic analysis [41] was conducted using an inductive approach, following Braun and Clark’s reflexive thematic analysis method [34]. Firstly, three researchers (C.L., C.V., M.V.) independently familiarised themselves with the data by reading all of the transcripts line by line. After 14 interviews, the researchers recognised the same themes occurring; after 16 interviews no new themes appeared, suggesting that data saturation [38] had been achieved. Then, two researchers (C.L., C.V.) coded each transcript inductively, affixing labels for quotations relevant to the study aim. After that, four researchers (C.L., C.V., M.V., I.M.) grouped the labels by similarity, first identifying subthemes, and gave each a name. All the researchers checked the categories that had emerged against the encoded data; one additional researcher (S.C.) checked the consistency of the data and some sections of the transcripts were re-read. Disagreements were resolved following consultation with a senior researcher (A.P.). At the end of this process, the whole research team categorised the final themes, also identifying one overarching theme; they reached full agreement on the themes. A trail code is shown in Table 1.

**Table 1:** Examples of the data analysis process: trail code

Quotations	Labels	Sub-themes	Themes
“Maybe I take it half an hour before or after, but I take it” (P1)	Early or late but I take it	Episodes of delays	Failure to systematically respect the rules
“This therapy [cortisone] is very heavy and I still don’t sleep more than two hours at night, every night” (P8)	Insomnia for steroid therapy	Managing the complex single and combined medications' side effects	Coping with the effects of the immunosuppressors
“I have a note [with medications] that I read every day because you have to read to avoid mistakes” (P3)	Follow written indications for medication intake	Setting the right strategies	Adopting personal strategies to become adherent

*P,* participant.

Member checking [38] was performed. Out of 16 participants, two (P7 and P10) were purposefully selected [37] as their responses were the most representative of the different recorded quotes that were relevant to the study aim. These two participants met in person with the principal investigator (C.V.) and the interview was audio-recorded. They agreed with the themes and subthemes that had been identified; therefore, no further interviews were needed.

### Ethical considerations

The study was approved by the Internal Review Board of the Department of Medicine, University of Udine, Italy (IRB Approval 166/2023, 11/09/2023). In accordance with the Declaration of Helsinki, written informed consent for study participation, audio-recording, and use of data was collected from participants. Participants were ensured anonymity and confidentiality. The narratives transcribed were anonymised immediately.

## Results

### Participants

The participants (*n* = 16, Table 2) were predominantly male (*n* = 10, 62.5%), had a mean age of 54.9 ± 15.3 years, and were married (*n* = 12, 75.0%). All reported having a caregiver who lived with them. Almost half of the participants reported a senior high school degree (*n* = 7, 43.7%) and being unemployed (*n* = 7, 43.7%). Allogeneic matched unrelated donor (MUD) human-leucocyte Antigen (HLA) 10/10 HSCT was performed in half of the participants (*n* = 8, 50.0%) and acute myeloid leukaemia was the most reported clinical indication for HSCT (*n* = 9, 56.2%).

**Table 2:** Demographic and clinical characteristics of participants (*n* = 16)

	***N* (%)**
Gender	
Male	10 (62.5)
Mean age ± SD [range] (years)	54.9 ± 15.3 [25–74]
Marital status	
Married Unmarried Divorced	12 (75.0) 3 (18.7) 1 (6.3)
With a caregiver	
Yes	16 (100.0)
Highest educational degree	
Senior high school University Middle school	7 (43.7) 5 (31.3) 4 (25.0)
Employment status	
Unemployed Employed Retired	7 (43.7) 5 (31.3) 4 (25.0)
Clinical indication for HSCT	
Acute myeloid leukaemia Myelodysplastic syndrome Others	9 (56.2) 3 (18.8) 4 (25.0)
Type of allogeneic HSCT	
MUD 10/10 Haploidentical HLA-identical	8 (50.0) 6 (37.5) 2 (12.5)
Grading of aGvHD [10] at diagnosis	
Grade I stage 2 skin Grade I stage 1 skin Grade II stage 3 skin Grade II stage 2 skin + stage 1 gastrointestinal Grade II stage 3 skin + stage 1 gastrointestinal Grade III stage 3 gastrointestinal	6 (37.5) 3 (18.8) 3 (18.8) 2 (12.5) 1 (6.3) 1 (6.3)
Mean onset of aGvHD ± SD [range] (days from HSCT)	26.9 ± 8.2 [18-46]
Onset of aGvHD during hospitalisation	
Yes	13 (81.2)
Mean time at the time of interview ± SD [range] (days from hospital discharge)	63.1 ± 58.8 [12–210]
Mean time at the time of interview ± SD [range] (days from aGvHD)	80.4 ± 56.2 [21–213]
Clinical response at the time of interview*	
Complete Partial Not valuable	12 (75.0) 2 (12.5) 2 (12.5)
Oral therapy at the time of interview	
Cyclosporine and prednisone Cyclosporine Prednisone Cyclosporine, prednisone and ruxolitinib	9 (56.2) 4 (25.0) 2 (12.5) 1 (6.3)
Photopheresis	
No	14 (87.5)

*aGvHD*, acute graft-versus-host disease; *HLA*, human-leucocyte antigen; *HSCT*, haematopoietic stem cell transplant; *MUD*, matched unrelated donor,* N*, number; *SD*, standard deviation.

*Extracted from medical records.

Regarding the onset of aGvHD, six (37.5%) participants developed grade II and one (6.2%) grade III with an average time of onset of 26.9 ± 8.2 days from HSCT that predominantly occurred during the in-hospital stay (*n* = 13, 81.2%). The interviews were performed on average 80.4 ± 56.2 days after the development of aGvHD and 63.1 ± 58.8 days after hospital discharge. Most participants reported a complete clinical response (aGvHD resolution; *n* = 12; 75%), and nearly half (*n* = 9, 56.2%) were in treatment with oral cyclosporine and prednisone. Only two patients (12.5%) underwent the photopheresis procedure.

### The perspectives of patients with aGvHD regarding MA to IMMs

**Fig. 1:**
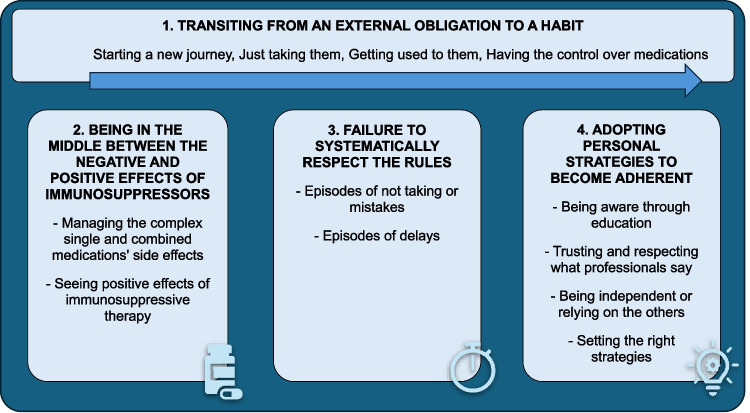
Main themes and subthemes summarising aGvHD patient’s perspective about MA to IMMs

Four themes emerged, including one overarching theme (Theme 1) as presented in Fig. 1 and Supplementary Table 3.

#### Theme 1: Transiting from an external obligation to a habit

This first overarching theme describes the perspective reported by patients on starting and assuming full control over their medications, where initially they faced difficulties and, in the end, taking medication became a habit. Overall, medications did not complicate or change the lives of participants (P1, P7), but the entire process leading to MA was a challenge.

After being discharged from the hospital, the majority of patients with aGvHD started a new journey compared to that already experienced after HSCT, with some initial difficulties leading to possible mistakes, because medications were so numerous that it was hard to understand what to take and when (P8). They did not feel confident about taking the right drug at the right time or were anxious when they saw the number of medications they needed to take at home for the first time (P14, P16). They also reported an unpleasant smell and taste of cyclosporine (P1, P13, P16); however, these difficulties were kept in check by a sort of compulsion to take medications (P1, P13). Patients reported that IMMs were considered an integral part of the GvHD and HSCT pathways; there was no other option except to have them (P2).

In this sense, patients just took their medication without thinking too much and with a sort of resignation (P3, P4, P14). As some of the patients were not used to taking many medications before HSCT, some patients with aGvHD perceived being forced to develop a new daily routine until they became used to it (P9, P11). Taking medications then became part of their everyday life, and a habit that marked their days (P15) until the patients assumed control over them (P8). One participant reported turning off the alarm before it sounded (P3), another ensuring that he did not run out of medication (P7); still others knew how to behaviour in case of forgetfulness (P6).

#### Theme 2: Being in the middle between the negative and positive effects of the immunosuppressors

During their journey, patients reported having lived in the middle between the negative and the positive effects of immunosuppressive therapy: they reported hand tremors (P4, P16) and hot flashes (P9) due to cyclosporine, or agitation and nervousness due to cortisone (P8, P15). One participant said that when she was upset, she jumped up “like a spring”, not recognising herself (P8). Insomnia was even reported (P8), which forced patients to “look at the ceiling for hours, failing to sleep” (P10); body and face swelling (P10, P12), hypertension (P13) and steroid-induced diabetes (P13, P15) were also experienced.

Also attributed to therapy were hirsutism, drowsiness, and “my marrow is very poor”— in this case, the use of other words instead of the medical term cytopenia (P7, P10). Foods or drinks, such as orange soda, took on a different flavour (P1), with an increased salty taste (P5) and they reported a mouth always kneaded or bitter (P16). Some patients believed that this condition was caused by taking many different pills during the day (P1, P3), whereas others believed that cyclosporine and cortisone caused it (P5, P13). One patient attributed hoarseness to cortisone (P15).

Despite this, patients continued to take the IMMs, thanks in part to the visible improvements in both cutaneous (P16) and intestinal aGvHD symptoms (P8, P10) and an increase in their appetite (P11, P14).

#### Theme 3: Failure to systematically respect the rules

To cope with the situation, patients shared their failure to systematically respect the rules of medication intake given in our centre (see “Study context”). Although they attempted always to take their medications, their MA was not consistent. Episodes of non-taking, mistakes or delays in medication management occurred, both intentional and unintentional.

One patient reported that sometimes he did not take his cyclosporine pills, and that this was “a delinquent act” (P6), so he seems to be aware that this is not good. Moreover, MNA episodes occurred due to health conditions such as “stomach issues and vomiting” (P2), mistakes such as reversing the schedules of two medications, (P16) or because some pills were not included in the weekly pillbox (P8). There were reports of IMMs not always being taken on time, such as taking them 30 min (P1) or an hour too early or too late (P15) relative to the expected time. In the participants’ opinion, the delay was minimal, so it did not matter too much and had no consequences (P10, P16).

#### Theme 4: Adopting personal strategies to become adherent

Patients adopted different strategies to ensure MA, such as receiving information from HCPs or asking for family support. Some participants reported knowing exactly what medications they were taking, including the dosage and the reason why (P5, P7), whereas others did not know why they were taking their medications (P3, P4). However, IMMs were “the main ones” to which priority was given, particularly cyclosporine (P5). Non-immunosuppressant medications were considered to be of secondary importance (P2). This information was probably given by HCPs; thus, HCPs play a crucial role in educating patients, thanks to the relationship that is established during hospitalisation that promotes a sense of trust in the patient (P3). Patients “rely on and trust absolutely, blindly” (P6) the HCPs’ knowledge and competence and the recommendations they provide.

Some patients reported that they managed their medication independently (P7); others required help and support from familiars and other caregivers (P1, P6, P7, P10). There were also some patients who completely relied on a caregiver (P3). The support received was both positive and negative: a friend of a participant (P10) reinforced the need to take medications after the transplant. In contrast, the partner of a patient (P1), who underwent an allogeneic HSCT years ago and was not adherent after discharge, provided a negative example of MNA.

Many strategies promoting MA have been reported, some aimed at remembering which medication to take and at what time, such as scrupulously reading (P16) and consulting the treatment plan (P3, P4) or a summary grid (P9, P15), also with the help of the caregiver (P3). Others preferred to write the time of intake directly on the medicine boxes (P1, P13) or to use alarm clocks or apps with notifications as reminders (P3, P5, P9, P14), including setting up a daughter’s mobile phone so that the alarm became a game time between father and daughter (P7). Patients shared the need to keep a stock of medications (P7, P8) to avoid the risk of being without and the need to ask the doctor for refills in advance. Carrying the medications with them when going out was also reported (P3, P13). Some patients associated medication intake with meals, taking the pills close to their plate or waiting and getting up from the table only after taking them (P8, P11, P12).

Two patients (P8, P10) prepared the required pills for the whole week on Sunday or Monday, putting the tablets in a pillbox with the days and hours of intake written on it. Finally, knowing that medication improves the health conditions was the thought guiding one patient to the right intake (P15). To ameliorate the taste and reduce the unpleasant smell of cyclosporine, patients took it with tea, milky coffee, soy drink, Coca-Cola or water with black cherry syrup (P1, P8, P15, P16), or they put it in the fridge before taking it (P10, P16). There were also those who reported “breathing with the mouth, rather than with the nose” to perceive less smell (P16).

## Discussion

To our best knowledge, this is the first study exploring patients’ perspective on MA to immunosuppressive therapy when diagnosed with aGvHD. The participants’ demographic profile was in line with that documented previously [42]; acute leukaemia and allogeneic MUD were the more common indications for and types of HSCT as mostly performed in Europe [1]. Most patients developed cutaneous manifestations during hospitalisation; therefore, they were aware of the symptoms and treatment medications required before their discharge. Cyclosporine and prednisone were the most commonly taken medications, in line with the cornerstones of first-line aGvHD therapy [2, 12].

Patients’ perspectives were summarised into four themes. The first was an overarching theme that synthesised the patients’ inner journey while transiting from seeing MA as an external obligation to seeing MA as a habit. The other three themes symbolised the steps experienced from the beginning of the ongoing treatment to its end, when patients achieved MA using personal strategies.

The first theme reiterates the initial difficulties in taking, timing and dosing while managing medications, as already reported in the literature on transplanted patients [31, 32]. MA in the implementation phase [23] is challenging for patients with aGvHD due to different factors, such as the smell of cyclosporine or the high number of pills. However, patients reported rightly being obliged to take medications, in a sort of resignation. This sense of obligation has not been reported among HSCT patients [26, 30–32, 39], and the clinical importance of IMMs in preventing the evolution of cGvHD [15] may have played a role. By creating a habit, patients develop control over medications, also becoming able to manage possible issues (e.g. missing a dose) independently without necessarily contacting the hospital, as done by a transplant patient who had forgotten to refill her medications on time and needed an immediate supply from the doctors [39].

The negative effects attributed to immunosuppressive therapy (e.g. change of taste [13], tremors [14]) have been documented in the literature. However, despite their negative implications for MA [14, 30], our participants seem to have coped with them positively. They might have internalised and accepted the possible negative effects of not taking IMMs, such as the risk of cGvHD, or they are simply burdened by having aGvHD. According to the Necessity-Concerns Framework [43], factors influencing MA include the perceived need for medications for current and future health conditions. This is in line also with research by Amato et al. [26] among allogeneic HSCT patients, and with research by Song et al. [30], where recalling the benefits of medications increased MA.

Episodes of MNA emerged in the third theme as mistakes, delays or isolated health issues preventing medication intake. Some examples mirrored those documented among allogeneic HSCT patients [14, 44]. However, regarding delays, the role of HCPs is impactful: as an example, lack of attention by nurses to patients’ forgetfulness justified a delay in taking the prescribed medications at the recommended time, thus implicitly transferring negative attitudes to the patient [45].

The risk of delaying or forgetfulness could also depend upon improvements in clinical conditions: patients with a mild form of a disease tend to be less adherent than patients with moderate severity [46]. Therefore, promoting not only educational but also behavioural interventions focused on taking IMMs on time and reducing moments of forgetfulness— also led by poor executive function after HSCT [47]—seems to be the priority in these patients. However, studies measuring the clinical impact of a delayed intake in the HSCT population (e.g. worsening aGvHD) are recommended because knowledge is related to the solid organ transplant setting [28].

As emerged in the fourth theme, patients used various personal strategies to develop MA. Firstly, they developed awareness through education, although in our centre there is not yet a systematic educational intervention regarding medication management. MA was reported when patients knew their medication regimen [39], but individual variability in what the patients want to know about their condition must be taken into account: they may enact a defence mechanism [20] or they may attribute responsibility to the caregiver as an external locus of control [26]. HSCT survivors are accustomed to searching for information on the internet [30, 32]; in our study, patients with aGvHD seemed to rely only on HCPs to receive information. However, support for emotional needs is also reported [39]. When provided, educational interventions should be based on a solid relationship between the HCP and the patient [48]. Fully relying on HCPs could be considered a double motivation: on the one hand, asking advice of HCPs could be an expression of the willingness to receive positive feedback as a kind of external reward (extrinsic motivation); on the other hand, it could be a vehicle to achieve the goal of being competent and independent in medication management when going home (intrinsic motivation). Motivation is one of the three elements at the centre of the Behavioural Change Wheel (BWC) [49], a system useful for identifying, understanding and explaining behaviours and influencing factors. Motivation, capability and opportunity are the factors to be taken into consideration when identify what needs to change in promoting behavioural interventions, such as a good MA to IMMs. Respect to capability [49], emerged barriers that could have threaten the physical capability were episodes of stomach ache or vomiting as not being physically able to swallow pills, while the knowledge and application of the correct intake/timing of IMMs or the notice/remember of taking IMMs are related to the psychological capability. The above-mentioned lack of a structured educational intervention in our centre in promoting MA and the forgetfulness in taking IMMs due to possible limitations in memory, concentration or attention were other examples of compromised psychosocial capability [49].

Furthermore, examples of social opportunity [49] referred to the patients’ social context that could influence MA. Beyond HCPs, patients were independent in their medication management (e.g. in their intake or refills) or they were used to relying on others, such as family members, who reminded them or monitored the regularity of their medication consumption in addition to providing emotional support [32]. A positive relationship between patients and caregiver(s) has been documented to increase MA [50, 51], suggesting that this relationship should be considered when assessing the situation and when delivering educational interventions; for example, by including the reference caregiver. Next to relatives and friends—both examples of sources to increase or decrease MA—the patients did not mention peer support (apart from P1, in which the partner had also had a transplant). However, evidence regarding peer support for the recipients of solid organs (sharing advice and concerns among other transplant recipients) was found to be useful [29]. Finally, no aspects related to the environmental context of the BWC emerged among the physical opportunity area (e.g. distance to clinic, no regular follow-up or issues relating to travelling) [49].

The overall strategies to promote MA used by participants in this study were consistent with the literature [30–32]. However, patients did not report the use of electronic monitoring to promote reminders and record intake [31]; similarly, they did not mention non-pharmacological interventions (e.g. cryotherapy and photo biomodulation) to deal with the dysgeusia [52]. With respect to the intake of liquids other than water or tea, it is not known whether these may have an impact on pharmacokinetics or pharmacodynamics. Patients seem to rely on well-documented strategies, less on those discovered more recently, suggesting the need to continue the dissemination of evidence with the aim of increasing their awareness of the wide range of available strategies, given that those already used in the context of HSCT may not be effective. However, all these interventions echo the behavioural change techniques proposed by the BCW [49]: problem solving, action planning, social support, reminders and prompts were used by our participants in an attempt to increase their MA. Another example of the systematic application of this model in the development and implementation of an intervention to support MA is the international, interdisciplinary and multicentre SteM-cell-transplantatIon faciLitated by the eHealth (SMILe) project, where an integrated model of care—e-health and a nursing care coordinator—is successfully been developed based on the BCW in aiming to achieve a behavioural change, also in the view of MA [27].

## Limitations

The study has several limitations. Firstly, the date of the interviews (from aGvHD onset) was different among participants, and this may have introduced a recall bias regarding the MA journey. Secondly, patients belonged to the same cultural context and the same transplant centre, suggesting that a more diverse population should be included in the future, potentially at an international level. Finally, according to the study design, no comparison has been conducted between the strategies used as allogenic HSCT patients and that further used (or not) as patients with aGvHD. This also should be considered in future studies.

## Conclusions

Becoming an aGvHD patient requires effort to ensure MA, considering the crucial role of IMMs. Patients start a new journey: after being forced to comply and some initial difficulties, they become adherent as a habit. While patients live with both the negative and positive effects of this therapy, some episodes of MNA, mainly represented by delays or mistakes, both intentional and unintentional, occur. Independently, patients rely on caregivers or HCPs for support in MA management, adopting different strategies. Thus, even in patients with aGvHD, MA is a complex behaviour, and it is often a challenge.

These results can help HCPs to understand how best to design tailored educational strategies for patients with aGvHD to maximise their MA to IMMs. In addition to education, which is just one behavioural change technique, centres and universities need to develop and implement other adequate behavioural change interventions to optimally support and help the patients to attain good MA.

## Supplementary Information

Below is the link to the electronic supplementary material.

Supplementary file1 (DOCX 42 KB)

## Data Availability

No datasets were generated or analysed during the current study.
