# Supplementary material for: Perspective on oral medication adherence among patients with acute graft-versus-host disease: a qualitative descriptive study
Source: Support Care Cancer. 2024 Sep 4;32(10):633. doi: 10.1007/s00520-024-08825-4 (PMC11374915; doi:10.1007/s00520-024-08825-4)
Supplement: Supplementary file 1 — Supplementary file1 (DOCX 42 KB) [file 520_2024_8825_MOESM1_ESM.docx]

**Supplementary table 1** The COnsolidated criteria for REporting Qualitative research (COREQ) checklist (2007) [35]

| **N.** | **Items** | | **Guide questions/description** | | **Section(s)** |
| --- | --- | --- | --- | --- | --- |
| **Domain 1: Research team and reﬂexivity** | | | | | |
| *Personal Characteristics* | | | | | |
| 1 | Interviewer/facilitator | | Which author/s conducted the interview or focus group? | | Methods: Data collection |
| 2 | Credentials | | What were the researcher’s credentials? E.g. PhD, MD | | Methods: Participants, Data collection, Data analysis (C.V. is a female RN, PhD student; C.L. is a female RN, BNS; M.V. is a female RN, MNS; I.M., S.C. and A.P. are female RNs, PhD) |
| 3 | Occupation | | What was their occupation at the time of the study? | | Methods: Data collection |
| 4 | Gender | | Was the researcher male or female? | | See “Credentials” of Supplementary table 1 |
| 5 | Experience and training | | What experience or training did the researcher have? | | Methods: Data collection |
| *Relationship with participants* | | | | | |
| 6 | Relationship established | Was a relationship established prior to study commencement? | | Methods: Data collection | |
| 7 | Participant knowledge of the interviewer | What did the participants know about the researcher? e.g. personal goals, reasons for doing the research | | Methods: Participants | |
| 8 | Interviewer characteristics | What characteristics were reported about the interviewer/facilitator? e.g. Bias, assumptions, reasons and interests in the research topic | | Methods: Data collection | |
| **Domain 2: Study design** | | | | | |
| *Theoretical framework* | | | | | |
| 9 | Methodological orientation and Theory | What methodological orientation was stated to underpin the study? e.g. grounded theory, discourse analysis, ethnography, phenomenology, content analysis | | Methods: Study design | |
| *Participants selection* | | | | | |
| 10 | Sampling snowball | How were participants selected? e.g. purposive, convenience, consecutive | | Methods: Participants | |
| 11 | Method of approach | How were participants approached? e.g. face-to-face, telephone, mail, email | | Methods: Data collection | |
| 12 | Sample size | How many participants were in the study? | | Results: Participants | |
| 13 | Non-participation | How many people refused to participate or dropped out? Reasons? | | Methods: Participants | |
| *Setting* | | | | | |
| 14 | Setting of data collection | Where was the data collected? e.g. home, clinic, workplace | | Methods: Data collection | |
| 15 | Presence of non-participants | Was anyone else present besides the participants and researchers? | | Methods: Data collection | |
| 16 | Description of sample | What are the important characteristics of the sample? e.g. demographic data, date | | Results: Participants, Table 2 | |
| *Data collection* | | | | | |
| 17 | Interview guide | Were questions, prompts, guides provided by the authors? Was it pilot tested? | | Methods: Data collection | |
| 18 | Repeat interviews | Were repeat interviews carried out? If yes, how many? | | NA | |
| 19 | Audio/visual recording | Did the research use audio or visual recording to collect the data? | | Methods: Data collection | |
| 20 | Field notes | Were ﬁeld notes made during and/or after the interview or focus group? | | Methods: Data collection | |
| 21 | Duration | What was the duration of the interviews or focus group? | | Methods: Data collection | |
| 22 | Data saturation | Was data saturation discussed? | | Methods: Participants | |
| 23 | Transcripts returned | Were transcripts returned to participants for comment and/or correction? | | Methods: Participants | |
| **Domain 3: analysis and findings** | | | | | |
| *Data analysis* | | | | | |
| 24 | Number of data coders | How many data coders coded the data? | | Methods: Data analysis | |
| 25 | Description of the coding tree | Did authors provide a description of the coding tree? | | Methods: Data analysis, Table 1 | |
| 26 | Derivation of themes | Were themes identiﬁed in advance or derived from the data? | | Methods: Data analysis | |
| 27 | Software | What software, if applicable, was used to manage the data? | | NA | |
| 28 | Participant checking | Did participants provide feedback on the ﬁndings? | | Methods: Data analysis | |
| *Reporting* | | | | | |
| 29 | Quotations presented | Were participant quotations presented to illustrate the themes / ﬁndings? Was each quotation identiﬁed? e.g. participant number | | Results: The perception of patients with aGvHD regarding MA to IMMs, Supplementary table 3 | |
| 30 | Data and ﬁndings consistent | Was there consistency between the data presented and the ﬁndings? | | Results: The perception of patients with aGvHD regarding MA to IMMs, Figure 1, Supplementary table 3 | |
| 31 | Clarity of major themes | Were major themes clearly presented in the ﬁndings? | | Results: The perception of patients with aGvHD regarding MA to IMMs, Figure 1, Supplementary table 3 | |
| 32 | Clarity of minor themes | Is there a description of diverse cases or discussion of minor themes? | | Discussion | |

*BNS*, Bachelor in Nursing Studies*; IMM*, immunosuppressant; *MA,* medication adherence; *MNS*, Master in Nursing Science; *NA*, not appropriate; *RN,* registered nurse.

**Supplementary table 2** Guide for the semi-structured interview

| Dear participant,  as the doctors told you, you have developed acute graft-versus-host disease, abbreviated in GvHD, a complication of the HSCT.  1.* Could you please tell me what medications were you taking at home to prevent GvHD?  2.* How did this medication affect your quality of life?  3a. * What are the treatments and medications you are now following to treat the onset of GvHD?  3b. ** Could you please tell me what are the treatments and medications you are following to treat GvHD?  4. How was the initiation of these medications?  5. What do you think when you are taking this therapy?  6. How does the medications you are taking affect your quality of life? For example, side effects, intakes during the day?  7. Can you tell me about positive and negative aspects, if any, of the management of this medication?  8. What strategies do you use for taking medications?  9. Could you describe an episode in which you forgot to take the medication or took it late? What is the reason for this episode?  10. Are there other aspects important to you regarding the taking of immunosuppressive therapy for GvHD that you would like to share?  Thank you very much for your time and important contribution to this study.  Have a nice day! |
| --- |

*GvHD*, graft-versus-host disease; *HSCT,* hematopoietic stem cell transplantation.

* question to be asked only to participants who developed acute GvHD after discharge.

** question to be asked only to participants who developed acute GvHD during hospitalization.

**Supplementary table 3** Themes, subthemes and main quotes

| **Themes** | **Subthemes** | **Quotes** |
| --- | --- | --- |
| *Transiting from an external obligation to a habit* | Starting a new journey | “At the beginning, I had some problems […] honestly because medications were so many that I had trouble understanding what to take and when. At the beginning, I skipped a line... medications were and are really many” (P8)  "At the beginning, I had a little anxiety… I took two or three at once..." (P14)  "At home, when I saw all those boxes, I was shocked because I didn’t remember I was taking all those drugs" (P16)  "The problem is just with cyclosporine that has a smell that I cannot cope with" (P1)  "[Cyclosporine] stinks [...]. If one has nausea and put that [...]. When you open the box, it makes you throw up" (P16)  "I have to take, it’s not that I can change it... which is prescribed I have to take" (P1)  "I have to take [medications], it’s not that I can change it... this was prescribed to me and this I have to take” (P13)  "You find in a choice, where there is no alternative" (P2) |
|  | Just taking it | "Just take them, with lots of water" (P3)  "I swallow, I don’t think about it too much" (P4)  “Just take it now” (P14) |
|  | Getting used to | "Then I got used to it..." (P9, P11)  "You take them at fixed times [...]. You have three moments: morning, noon and evening" (P15)  "More than having to take [cyclosporine] once in the morning and once in the evening, I don’t have to do anything else" (P1)  "From a psychological point of view, all these medications [...] do not complicate my life or my daily existence" (P7) |
|  | Having the control over medications | "The situation is quite over control” (P8)  "For the moment, since I am always at home, I turn them off [the alarm clocks] before they ring" (P3)  "However, as regards the handling of pills and their refilling [...], it is appropriate that I do not find myself without them" (P7)  "If I do not remember to take a tablet at 4 o’clock, I take it at 6" (P6) |
| *Being in the middle between the negative and positive effects of the immunosuppressors* | Managing the complex single and combined medications’ side effects | "This tremor is due to cyclosporine" (P16)  "At the beginning, when I took it [cyclosporine], I got hot flashes [...]. It happens to me also sometimes after eating" (P9)  "Cortisone is a drug that makes me quite, quite nervous... I am very nervous..." (P15)  "The moment I feel upset or not considered, I see that I “jump up” ... like a spring, this is not me. I think prednisone gave me this kind of reaction" (P8)  "With cortisone, I slept very little, I was wide-eyed, moreover because I could not take drops to sleep having had seizures. I spent nights looking at the ceiling" (P10)  "Cortisone just inflates me, the doctors have reduced and now I am less swollen" (P12)  "I already had cortisone in the hospital, which unfortunately raised my pressure and gave me diabetes” (P13)  "The immediate effect [of cortisone] is the lowering of the voice, I am hoarse… this is not my voice..." (P15)  "I know that immunosuppressants give hirsutism, so at this moment everything is growing, and this also grows [she laughs pointing the chin]" (P10)  "Sometimes, they [the medications] seem to have a drowsy effect..."(P7)  "Actually, I am taking cortisone and cyclosporine and ruxolitinib; taking these three, my marrow is very poor, because it is full of drug toxicity and therefore cannot produce cells" (P10)  "[The medications] made me change the taste. Many things are different, for example orange juice, I feel another taste, it is no longer a beautiful sweet" (P1)  "Now I’m feeling [the taste], after cyclosporine [...]. I feel the salty three times as much as before. All the medications as side effect have taste disorders" (P5)  "My mouth is always bitter and it’s due to drugs" (P16)  "When I eat, I don’t feel food tastes because I always have my mouth kneaded... I take 10 medications" (P3)  "I still don’t feel the taste very well [...]. They told me that it is normal [...], it is due to the therapy I did even after, immunosuppressants, antiviral..." (P13) |
|  | Seeing positive effects of immunosuppressive therapy | "But it didn’t last very long [the itching], that was the good thing... medication probably worked right away" (P16)  “Now at home it’s much better, I’ve only taken loperamide once in the last few weeks. It’s been getting much better” (P8)  "[Ruxolitinib] to treat intestinal GvHD is fortunately working, because it may not even work and have to treat it with other methods" (P10)  "[Cortisone] helped me with appetite" (P11) |
| *Failure to systematically respect the rules* | Episodes of non-taking/mistakes | “[It happened not to take] cyclosporine, even though I know it was a delinquent act” (P6)  "It happened to me twice that unfortunately I started eating, I threw up and I missed the therapy, just twice because I was sick" (P2)  "One afternoon that I was sick with my stomach, I was not well... I took the one of 8 o’clock at 6, and the one of 6 o’clock I took it at 8" (P16)  "I don’t forget [to take medications], it just happens because there are three pills [...] that don’t fit in the pillbox. So sometimes I forget them, once a week, I lose them and... what happened" (P8) |
|  | Episodes of delays | "Maybe I take it half an hour before or after, but I take it" (P1)  "Maybe I’m over half an hour, but I think it doesn’t matter too much" (P2)  "An hour or so less... I don’t think it will change, swing half an hour or an hour" (P4)  "If you tell me an hour’s delay... maybe" (P15)  "I like to sleep so instead of 8 o’clock maybe I take it at 09.30 or 10 o’clock" (P5)  "When you go home, honestly, sometimes it happens to delay [therapy] for a couple of hours. If you take them once a day and change the time intake… nothing happens" (P5)  "I’m not really precise with schedules [...] but in my opinion it does not change if you take it an hour or half an hour late" (P10)  "Nothing happens for an hour, I think..." (P16) |
| *Adopting personal strategies to become adherent* | Being aware through education | "I take cyclosporine and cortisone, before it was 50 mg and now 25 mg. They decided to remove cyclosporine and leave cortisone" (P5)  "There were drugs [for GvHD] ... as cortisone. I started with three tablets a day and then to climb, until I reached half a tablet, which I interrupted on September 30" (P7)  "[The reason why I take cyclosporine] I don’t know... today doctors reduced it and I don’t know why... I don’t know why I’m taking it" (P3)  "These are not questions for me, I have no idea" (P4)  "The important thing is to stay in the therapeutic levels, especially for cyclosporine. The other drugs... cyclosporine is the most important" (P5)  "[For GvHD] I checked with the doctors and nurses. It seems to me that they [cyclosporine and cortisone] were those who controlled everything" (P2)  "I was told that when you take orally [cyclosporine] you will have to put it in the fridge first" (P10) |
|  | Trusting and respecting what professionals say | "I always respected what the doctors told me, at what time and what medicine, respected in full all the times" (P3)  "I rely on and trust absolutely, blindly, I do not even think about inventing something, they [professionals] studied and are good... I do what they tell me to do" (P6) |
|  | Independently or relying on the others | "I did always everything on myself" (P7)  "My mother reminds me that I have to take [pills], even my partner sticks me" (P1)  "There is my wife and then I do it on my own" (P6)  "[My parents] don’t feel sure to leave me alone, so I’m always with them, they are very close to me" (P10)  "My daughter follows everything... I don’t know... she tells me to take those pills and I take them" (P3)  "A friend of mine, who did the transplant, told me that he, as soon as he got out of hospital, was taking a lot [of drugs] ..." (P10)  "My partner [she underwent a HSCT five years ago] did the first period after discharge in which she didn’t even take the pills, she hid them. Then she had various problems... if you do not take the tablets..." (P1) |
|  | Setting the right strategies | "I sit down, I have the sheet [of the medication regimen] and I start with... this, this, this. Then I check it again and I tell myself if I took one of these, one of these... Then I reconvene, then I recount it" (P16)  "I have a note [with medications] that I read every day because you have to read to avoid mistakes" (P3)  "We have the schedules written on a sheet that I always consult in order not to make mistakes" (P4)  "I made a scheme, the therapy of 8.00, 12.00, 16.00 and for the evening. I cross [on a sheet] [...]. Then, since there are many of them, the one of 16.00 I remember but, in any case, I’ll check" (P9)  "My daughter made me a grid with at 7 o’clock the tablets that I have to take, at 8 the others, at 12 this, at 16 this and at 20 these. I respect it [...], we made a summary" (P3)  "On the pill box, I write when I have to take them. I just remember" (P1)  "[Alarm clock] I put it at 8, 12, 16 and 20 on my mobile phone" (P3)  "I remember to take medications, possibly using the alarm clocks" (P5)  "I also have the app on my phone [...]. On that I put the reminder just for the tablet of 4 o’clock because maybe I’m busy and I get lost" (P9)  "I have a reminder on my phone. Especially for the cyclosporine you have to take at the same time" (P14)  "At home it has become a game with my daughter [...]. What happened was that I asked her to set the alarm and, if the alarm sounded [on her mobile] and she was somewhere else, she came and reminded me that it was time to take the pills" (P7)  "I also take stock when I have to come here, so they give me the new box or the prescription" (P8)  "If I’II go out, I take with me water and tablets… at 4 o’clock where I am, I stop and take the pill" (P3)  "I carry around the single blister in the day. Since I have connected medications to meals, I take them immediately after breakfast, right after lunch. I don’t need a reminder" (P8)  "I have a schedule: when I eat, on a full stomach, as it is written there. If at 8 am I have breakfast, at 8.30 I take them" (P11)  "I prepare the tablets once a week, usually on Saturday evening or Sunday morning, I prepare the pillbox for the whole week" (P8)  "What is the thought? That medications can lead me to an improvement of my condition" (P15)  "I drink them with tea, or, in the morning, I take it with milky coffee, with a little of sugar just to remove that taste" (P1) "I take the tablets with water but in the morning, at breakfast, even with a soy drink, because I noticed that it covers a lot of flavours and I can take several at a time" (P8)  "I immediately drink something [taking cyclosporine]... water or maybe some Coca Cola sometimes, usually water" (P15)  “I cannot drink water... I have a thermos, today I took a bit of milk-coffee or water with black cherry or tea. The important thing is that it has some flavour because the mouth is always bitter" (P16)  "The nurses tell you: ‘I give it to you, you put it 15 minutes in the fridge and then you take it’" (P10)  "I don’t breathe with the nose; I breathe with the mouth, so I don’t smell it" (P16) |

*GvHD*, graft-versus-host disease*; HSCT,* hematopoietic stem cell transplant; *P*, participant.
